# Atherosclerosis and Toll-Like Receptor4 (TLR4), Lectin-Like Oxidized Low-Density Lipoprotein-1 (LOX-1), and Proprotein Convertase Subtilisin/Kexin Type9 (PCSK9)

**DOI:** 10.1155/2024/5830491

**Published:** 2024-02-27

**Authors:** Bahador Bagheri, Zahra Khatibiyan Feyzabadi, Ahmad Nouri, Ali Azadfallah, Mahyar Mahdizade Ari, Maral Hemmati, Mahboubeh Darban, Parisa Alavi Toosi, Seyedeh Zahra Banihashemian

**Affiliations:** ^1^Cancer Research Center, Semnan University of Medical Sciences, Semnan, Iran; ^2^Center for Molecular Cardiology, University of Zurich, Schlieren, Switzerland; ^3^Student Research Committee, Faculty of Medicine, Semnan University of Medical Sciences, Semnan, Iran; ^4^Department of Internal Medicine, Kowsar Hospital, Semnan University of Medical Sciences, Semnan, Iran

## Abstract

Atherosclerosis is a leading cause of death in the world. A significant body of evidence suggests that inflammation and various players are implicated and have pivotal roles in the formation of atherosclerotic plaques. Toll-like receptor 4 (TLR4) is linked with different stages of atherosclerosis. This receptor is highly expressed in the endothelial cells (ECs) and atherosclerotic plaques. TLR4 activation can lead to the production of inflammatory cytokines and related responses. Lectin-like oxidized low-density lipoprotein-1 (LOX-1), an integral membrane glycoprotein with widespread expression on the ECs, is involved in atherosclerosis and has some common pathways with TLR4 in atherosclerotic lesions. In addition, proprotein convertase subtilisin/kexin type9 (PCSK9), which is a regulatory enzyme with different roles in cholesterol uptake, is implicated in atherosclerosis. At present, TLR4, PCSK9, and LOX-1 are increasingly acknowledged as key players in the pathogenesis of atherosclerotic cardiovascular diseases. Herein, we presented the current evidence on the structure, functions, and roles of TLR4, PCSK9, and LOX-1 in atherosclerosis.

## 1. Introduction

### 1.1. Atherosclerosis

Atherosclerosis is one of the most common causes of cardiovascular diseases (CVDs) worldwide and a common cause of death in the United States of America, Europe, and Japan [1, 2]. Smoking, hypertension, dyslipidemia, and diabetes mellitus are the major risk factors for atherosclerosis [3]. Endothelial dysfunction is a hallmark in the pathogenesis of atherosclerosis. One of the early stages of atherosclerosis is vascular wall damage, accompanied by changes in arterial permeability [4]. Disruption in arterial permeability results in loss of vascular polarity and makes the endothelial cells (ECs) spindle-shaped; this process is the reason why endothelial dysfunction and plaque formation usually occur in areas with stressful hemodynamic states such as arteries with high curvature or arterial branches with turbulent blood flow [5, 6] Furthermore, adhesion molecules like vascular cell adhesion molecule-1 (VCAM-1), intercellular adhesion molecule-1 (ICAM-1), E-selectin, and P-selectin facilitate the diapedesis of leukocytes and the inflammatory response [7–10].

### 1.2. Lipid Accumulation

**Figure 1:**
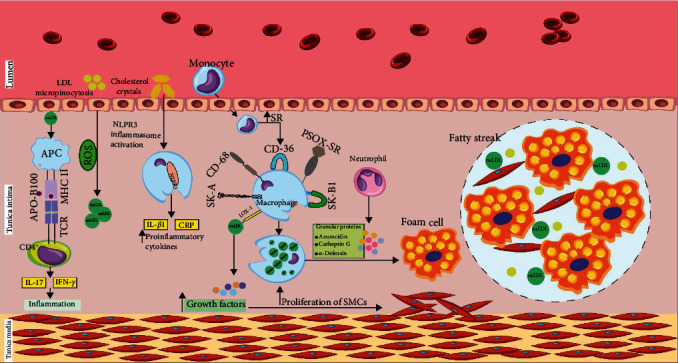
Different players in atherosclerosis. Increased LDL enters the cell via micropinocytosis and is converted into oxLDL. Additionally, after digesting oxLDL by APC, TCRs detect APO-B100 as an autoantigen by MHC2, leading to the release of IL-17 and IFN-*γ*. Absorbed cholesterol crystals can activate NOD, LRR, and pyrin domain-containing protein 3 (NLRP3) inflammasome, causing the production of IL-1*β*. Furthermore, monocytes pass through the endothelium and differentiate into scavenger receptors, including SK-A, SK-B1, CD36, CD68, LOX-1, and SR-PSOX. Macrophages will be enabled to absorb more oxLDL. Meanwhile, granular proteins such as azurocidin, cathepsin G, and *α*-defensin produced by neutrophils, facilitate the conversion of macrophages to foam cells. Additionally, the binding of oxLDL to LOX-1 leads to the release of growth factors that can increase the proliferation of smooth muscle cells. In summary, the aggregation of foam cells can create fatty streaks, which is the initial step in the formation of atherosclerotic plaque. LDL, low-density lipoprotein; oxLDL, oxidized low-density lipoprotein; APC, antigen-presenting cell; TCRs, T-cell receptors; APO-B100, apolipoprotein B-100; MHC2, major histocompatibility complex class II; IL-17, interleukin-17; IFN-*γ*, interferon-gamma; NLRP3, NOD-, LRR-, and pyrin domain-containing protein 3; IL-1*β*, interleukin-1*β*; SK-A, scavenger receptor class A; SK-B1, scavenger receptor class B type 1; CD36, cluster of differentiation 36; CD68, cluster of differentiation 68; LOX-1, lectin-like oxidized low-density lipoprotein receptor-1; SR-PSOX, scavenger receptor that binds phosphatidylserine and oxidized lipoproteins.

During the early steps in atherosclerotic plaque formation, the LDL particles undergo oxidation, which is influenced by reactive oxygen species (ROS). The LDL particles will subsequently bind to scavenger receptors (SRs) on macrophages to produce foam cells with abundant amounts of lipid reserves [5, 8, 11–13]. The LDL uptake into the macrophages can be done by micropinocytosis or by phagocytosed crystals [11]. The formation of fatty streaks from the accumulation of LDL-oxidized particles in foam cells is a characteristic of the early stages of atherosclerosis [8, 9] (Figure 1).

As monocytes cROS the endothelium, they become tissue macrophages, resulting in the differentiation of a series of SRs such as SK-A, SK-B1, CD36, CD68, lectin-like oxidized low-density lipoprotein-1 (LOX-1), and SR-PSOX, which enable them to pick up the oxidized LDL and phosphatidylserine particles [7, 8, 10, 11]. Cholesterol particles adsorbed by activating the NLRP3 (NOD-, leucine-rich repeat (LRR)-, and pyrin domain-containing protein 3) nuclear pathway cause the production of pro-inflammatory cytokines like IL-1*β* and C-reactive protein (CRP), accompanied by a gradual increase in the levels of prostaglandins, matrix metalloproteinases (MMP), nitric oxide species (NOS), and ROS [11, 14, 15]. After digestion of LDL oxidized particles by antigen-presenting cell (APC), the Apo-B100 component is detected as an autoantigen by the MHC-II molecule via T-cell receptor (TCR) of CD4+ T lymphocytes [6, 11, 14]. Furthermore, neutrophils, under innate immunity, can exacerbate the atherosclerosis process by producing and secreting granular proteins such as azurocidin, cathepsin G, and *α*-defensin; these cells can also facilitate the conversion of macrophages to foam cells [7]. In advanced atherosclerotic lesions, mast cells secret histamine, serotonin, leukotriene, thromboxane, serine protease, and other eicosanoids, which may lead to plaque rupture [7, 14], especially in the presence of metalloproteinases [16]. Growth factors are secreted from the endothelium, smooth muscle cells (SMCs), and macrophages in response to OxLDL and LOX-1 interaction, which may lead to smooth muscle proliferation, extracellular matrix production, and the formation of new arteries [17]. It is important to note that growth factors are released under specific circumstances, particularly when levels of oxidative stress are low [18]. These new and unstable arteries increase the risk of intraplaque bleeding and subsequent atherosclerotic plaque rupture [5, 6, 8, 10, 19].

### 1.3. Complications of Atherosclerotic Plaques

In general, plaques may go into a nonobstructive, asymptomatic, and prolonged state over several months to years, which may progress toward plaque rupture or chronic vascular occlusion [14, 20]. In the extracellular matrix of the plaque, there is a balance between reconstruction and degradation depending on factors such as necrotic nucleus size, temperature, or pH of the environment tissue [5]. In general, it is estimated that up to 65% of plaques ultimately lead to cap rupture, 30%–35% lead to superficial erosion, while only 2%–7% of them remain stable and lead to calcification [10].

Some plaques only lead to erosion or localized damage to the endothelium without the plaque rupturing. Areas with a high risk of erosion have high levels of smooth muscle tissues and proteoglycans and low levels of macrophages. Since surface vulnerability increases endothelial destruction, there may be an increase in aggregation of the platelets in the eroded areas, which may eventually lead to thrombosis formation [5, 14].

## 2. Toll-Like Receptor 4 (TLR4)

### 2.1. Structure, Function, and Signaling

TLRs were first discovered in the dorsal–ventral development of drosophila in 1997 [21]. TLR4 is a type 1 transmembrane protein that contains an LRR extracellular domain and a carboxy-terminal intracellular domain similar to the intracellular domain of the interleukin 1 receptor [22]. As shown in Table 1, TLR4, similar to the other TLRs in its family, can recognize different types of exogenous pathogen-associated molecular patterns, such as lipopolysaccharide (LPS). Likewise, several endogenous ligands for TLR4 have also been discovered, including domain A fibronectin (Fibronectin-EDA) and heat shock proteins.

**Figure 2:**
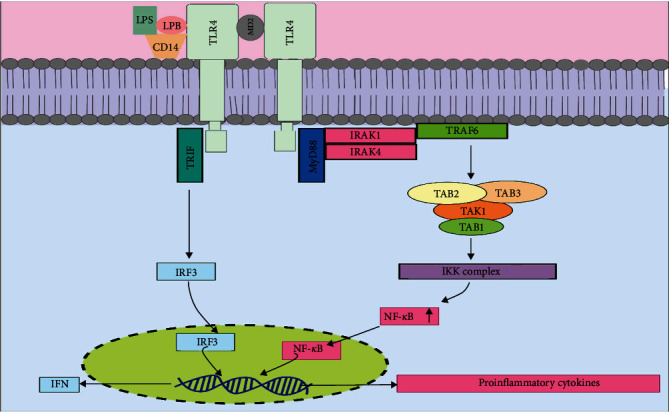
TLR4 signaling cascade. Circulating LBP recognizes LPS in the plasma and brings it to CD14. This aids the loading of LPS onto the LPS receptor complex, which is composed of dimerized TLR4 receptors and two molecules of the extracellular adapter MD-2. Subsequent signals activated by TLR4 can be subdivided into those dependent on MyD88, which occur early, and those independent of MyD88, which occur later and use the adapters TRIF and TRAM. LPS signaling leads to the early activation of NF-*κ*B, IRF3, and MAPK kinase pathways, which are mediated by MyD88. After the subsequent activation and phosphorylation of IRAK, TRAF6 becomes activated, which gives rise to the expression of numerous pro-inflammatory genes. As a later response to LPS, TLR4 gives rise to the activation of TRAF6 and TBK1, an event mediated by the adapters; TRIF and TRAM. LPS, lipopolysaccharide; LBP, LPS-binding protein; MyD88, myeloid differentiation factor 88; IRAK, IL-1R-associated kinase; TLR, Toll-like receptor; TRAF, tumor necrosis factor receptor-associated factor; TRIF, Toll/IL-1R domain-containing adaptor-inducing IFN-*β*; IKK, inhibitor of nuclear factor-*κ*B (I*κ*B) kinase.

Palsson-McDermmot and O'Neill [23] explained the downstream signaling cascade of TLRs entirely in 2008. TLR4 expressed on the surface of hematopoietic and nonhematopoietic cells such as ECs is in noncovalent association with myeloid differentiation 2 (MD2). This association is required for ligand-induced activation and forms the TLR4/MD2 receptor complex. Upon LPS recognition that leads to several molecular interactions, including LPS-binding protein (LBP), CD14, MD-2, and TLR4 and oligomerization of TLR4, downstream adaptors such as myeloid differentiation factor 88 (MyD88) are recruited through interactions with the Toll-interleukin-1 receptor (TIR) domains [24]. However, TLR4 mainly acts through MyD88-dependent and MyD88-independent pathways. In response to MyD88 binding, IL-1R-associated kinase 1 (IRAK1) is activated due to phosphorylation of IRAK1 by IL-1R-associated kinase 4 (IRAK4). This allows tumor necrosis factor-associated receptor 6 (TRAF6) to bind the phosphorylated IRAK4-IRAK1 complex. The second complex, TAK1-binding protein 1 (TAB1), TAK1-binding protein 2/3 (TAB2/3), is activated when IRAK1–TRAF6 dissociates from the TLR4 (Figure 2). When TAK1 is activated, it stimulates the inhibitor of nuclear factor-*κ*B (IkB) kinase complex (IKK complex), which then phosphorylates IkB proteins, which causes its degradation. After that, due to the translocation of NF-*κ*B to the nucleus, the production of several pro-inflammatory cytokines is promoted. In the independent myD88 pathway, upon activating TLR4, TIR domain-containing adapter molecules (TRIF or TICAM-1) bind to the intracellular TIR domain. TRIF activates IFN-related factor 3 (IRF3), which then activates the transcription of target genes like interferons [22, 23].

### 2.2. TLR4 and Atherosclerosis

The critical role of TLRs in atherosclerosis is well-documented. In addition to its role in pathogen recognition, TLR4 is expressed by a variety of cells in atherosclerotic lesions. Although TLR4 is expressed at low levels by ECs in normal arteries, Edfeldt et al. [25] reported increased expression of TLR4 on the ECs of human atherosclerotic lesions. Vascular smooth muscle cells (VSMCs) that reside in the media of healthy adult arteries and regulate vascular tone seem to upregulate TLR4 expression in human atherosclerotic vessels [26]. It was found that TLR4 is expressed in adventitial fibroblasts at the site of the formation of intimal lesions [27]. Dendritic cells also mediate the immunity-related processes of atherogenesis development through cell–cell contact with innate and adaptive immune cells [28]. Platelets are involved in the atherosclerosis process. Activated platelets can release platelet microparticles, which are highly procoagulant [29, 30]. Additionally, TLR4 is expressed on platelets, and activation of platelets by LPS triggers coagulation via TLR4 [31, 32]. High mobility group box 1 protein (HMGB1) is involved in the activation of platelets and plays a role in coagulant dysfunction during hemorrhagic shock and resuscitation [33]. Ahrens et al. [34] showed that in human coronary artery thrombi, the level of HMGB1 expression was increased. A growing body of evidence has shown that the formation of foamy macrophage cells through the interaction between activated monocytes and oxidized LDL (oxLDL) plays the main role in the development and progression of atherosclerosis. These lipid-laden foamy macrophages form the basis of the primary lesion. However, TLR4 also plays a significant part in this process by affecting the oxLDL-induced differentiation of macrophages to foam cells alongside the induction of inflammatory cytokine expression in VSMCs [35].

In the early stage of atherogenesis, activation of ECs and their overexpression of adhesive molecules leads to the rolling of circulating monocytes along the vascular surface and subsequent adherence at the site of activation. However, the LPS-induced activation of TLR4 on macrophages initiates a signal cascade that leads to the production of ROS and cytokines [25]. Methe et al. [36] found that acute myocardial infarction (MI) and unstable angina are associated with enhanced expression and signaling events downstream of TLR4 in circulating monocytes. Satoh et al. [37] reported a strong association between activation of TLR4 and heart failure following MI. The role of TLR4 in atherosclerosis is also supported by several loss-of-function animal models. According to Coenen et al.'s [38] study, deficiency of TLR4 expression in mice macrophages could reduce atherosclerotic lesion size under fed low-fat diets. Zeng et al. [39] found that treatment of high-fat fed ApoE−/− mice with intermittent hypoxia triggered the activation of pro-inflammatory TLR4/NF-*κ*B signaling, leading to accelerated growth and vulnerability of atherosclerotic plaque. Malgor et al. [40] also reported an overexpression of Wnt5a in coincident with TLR4 and TLR2 in an advanced stage of atherosclerosis.

Several studies have demonstrated the role of TLR4 in plaque rupture. Activated macrophage cells within the plaque degrade extracellular matrix by secretion of MMP and proteolytic enzymes, which lead to plaque rupture. Recognition of LPS by TLR4 induces the expression of MMP9 in human macrophages, which degrades the collagen of fibrous caps [41]. Induction of apoptotic molecules such as Fas–Fas ligand is another important event in plaque rupture [42]. Destabilization of plaque may also occur through the induction and activation of proteolytic enzymes *via* TLR4 in macrophages. Proteolytic enzymes are capable of degrading the components of the extracellular matrix and predispose plaque to rupture [43]. Together, these results suggested a pivotal role for TLR4 in atherosclerosis progression. Our previous investigations have provided evidence for TLR4 role in human atherosclerosis and associated complications [44–46]. We showed that there is an increase in monocyte expression of TLR4 in patients with chronic coronary syndrome (CCS) who underwent percutaneous coronary intervention (PCI) [45]. In another study, we found that thrombolytic (fibrinolytic) therapies caused a more increase in monocyte expression of TLR4 expression and function compared to PCI in patients with acute coronary syndromes (ACS) [44]. Moreover, it was shown that 100 mg hydrocortisone prior to PCI was effective to cause a reduction in TLR4 expression and function in patients with CCS [47].

## 3. LOX-1

### 3.1. Structure, Function, and Signaling

LOX-1 (also known as SR-E1) is a type II integral membrane glycoprotein that is encoded by the lectin-like oxidized low-density lipoprotein receptor 1 (OLR1) gene located on chromosome 12. This receptor belongs to the C-type lectin family and is the major receptor of oxLDL. LOX-1 consists of four domains, including a short N-terminal cytoplasmic domain, a transmembrane domain, an extracellular stalk region (neck domain), and a C-type extracellular lectin-like domain (which is responsible for binding to ligands, especially oxLDL) at the C-terminus [48]. Several studies indicate the expression of LOX-1 in different types of cells like ECs, SMCs, macrophages/monocytes, platelets, fibroblasts, cardiomyocytes, airway epithelial cells, renal, and neuronal tissues [49–55]. The ligands that have been introduced for this receptor include modified lipoproteins (oxLDL, acetylated LDL, and hypochlorite-modified high-density lipoprotein), bacteria, apoptotic cells, advanced glycation end-products (AGEs), activated platelets, polyinosinic acid, carrageenan, phosphatidylserine, phosphatidylinositol, and CRP [55–60]. Importantly, LOX-1 is also expressed in atheroma-derived of human and animal atherosclerotic lesions. The expression of LOX-1 is induced by several pro-inflammatory cytokines (TNF-*α*, IL-1, IFN-*γ*), CRP, LPS, modified lipoproteins, hypertension-related stimuli (angiotensin II, endothelin-1, and fluid shear stress), hyperglycemic stimuli (high glucose and AGEs), IL-6, and some other stimuli-like homocysteine and free radicals [48, 61–63]. Pathological conditions such as diabetes mellitus, hypertension, hyperlipidemia, myocardial ischemia, and atherosclerosis are associated with an induction in LOX-1 expression. LOX-1 is involved in OxLDLs/LDLs transcytosis, leading to the macrophages transformation to foam cells and proliferation of SMCs [64].

### 3.2. Influence of LOX-1 on ECs

ECs activation by the LOX-1/oxLDL axis, which tends to endothelial dysfunction, is a hallmark of atherosclerosis which leads to the reduced endothelium-dependent relaxation, increased monocyte adhesion to ECs, facilitates foam cell formation, and apoptosis of ECs [65].

Endothelial dysfunction is partially a consequence of oxLDL/LOX-1 interaction. Several signaling pathways play a part in this process. One pathway involves the production of ROS caused by increased activity of nicotinamide adenine dinucleotide phosphate (NADPH) oxidase. ROS, especially superoxide, impairs endothelial NO synthase (eNOS), which is responsible for producing nitric oxide using L-arginine and oxygen [66]. Meanwhile, LOX-1 directly increases L-arginase-1, which metabolizes arginine into ornithine and urea. As a result, there is a further decrease in NO levels and increased levels of inactivated NO. Finally, depletion of NO can lead to EC impairment [67]. Another pathway is related to EC apoptosis, which can enhance SMC proliferation and coagulation. OxLDL and LOX-1 can work together to activate the apoptosis pathway and deactivate the anti-apoptosis pathway; for example, they can increase caspase-3 and caspase-9 and decrease Bcl2 (an antiapoptotic protein) [68]. Lastly, oxLDL has a destructive effect on endothelial progenitor cells (EPCs), which disturbs the migration and proliferation of EPCs [69], and the resulting EPC dysfunction may play an important role in atherogenesis.

Leukocyte adhesion to the ECs is a crucial step in the development of atherosclerosis. Li and Mehta [70] demonstrated that incubation of human coronary artery ECs (HCAECs) with oxLDL results in the upregulation of monocyte chemoattractant protein-1 (MCP-1) expression, beside, using a human LOX-1 antisense RNA could inhibit this response. It suggests that LOX-1 is a key factor in ox-LDL–mediated monocyte adhesion to HCAECs [70]. Moreover, the binding of oxLDL to LOX-1 activates the NF-*κ*B signaling pathway and promotes monocyte adhesion to ECs [61].

### 3.3. LOX-1 and Atherosclerosis

LOX-1 is a cell surface SR that participates in the binding, endocytosis, and proteolytic degradation of oxLDL and also mediates the induction of endothelial dysfunction, vascular inflammation, foam cell formation, and collagen deposition, resulting in atherosclerosis [71]. Previous studies have shown that LOX-1 is overexpressed in atherosclerotic lesions. Inoue et al. [72] showed the overexpression of LOX-1 in atherosclerosis in a mice model. Expression of LOX-1 is mainly regulated through a feed-forward system stimulated by oxLDL, a major component of atherosclerosis. Several signaling pathways are induced by LOX-1, which leads to the activation of protein kinase, transcription factors, and regulation of apoptotic and antiapoptotic genes; the final result is the development of atheroma. In pro-inflammatory states, there is an increase in the expression of LOX-1 up to 40% [73]. Internalization of oxLDL into ECs by LOX-1 increases in the macrophages. In this process, calpains, which is a calcium-dependent protease, have a crucial role in macrophage migration [74]. Wang et al. [75] showed that macrophage migration is associated with upregulation of LOX-1 and calpain-2 and downregulation of calpain-1, the same as oxLDL. As a result, macrophages remain in the intima layer of arteries and then transform into foam cells, promoting the development of plaques. Eto et al. [76] demonstrated that after vascular injury, the expression of LOX-1 increases [76]. Overproduction of oxLDL is responsible for the upregulation of LOX-1 on SMCs, which triggers pro-apoptotic pathways in vascular SMCs [77]. Exposure to high levels of oxLDL can induce upregulation of pro-apoptotic protein Bcl-2-associated X protein (Bax) and also downregulation of antiapoptotic B-cell lymphoma 2 (Bcl- 2). This process may have an impact on vulnerability and rupture of the atherosclerotic lesions [78].

OxLDL/LOX-1 activation and enhancement of NADPH oxidase may lead to the increased production and activation of mitogen-activated protein kinase (MAPK) and transcription factors (such as NF-*κ*B), which can elevate the production of ROS and reduce the production of NO, ultimately leading to apoptosis and autophagy (autophagy refers to the destruction of cytoplasmic components by lysosomes, which differs from endocytic degradation by extracellular proteins and plasma membranes and is performed by the autophagosome [79]). Ding et al. [80] showed the dose-dependent VSMC's behavior in response to oxLDL level; a modest concentration (20–40 *μ*g/ml) caused autophagy and apoptosis, and a higher concentration (60–100 *μ*g/ml) caused apoptosis and declined autophagy. In vivo, it has been demonstrated that deletion of LOX-1 in low-density lipoprotein receptor (LDLR)/LOX-1 double-knockout mice alleviated autophagy [81]. Noncoding RNA, specifically microRNA, negatively modulates gene expression via binding to their mRNA [82]. Hsa-let-7g is a microRNA that can diminish the expression of LOX-1 and ROS formation in VSMCs [80]. It can also cause an overexpression of autophagy markers (beclin-1, LC3, and Atg5). Hence, there is a similar effect between hsa-let-7g and LOX-1 antibody.

Colocalization of LOX-1 and oxLDL within SMCs of human restenotic plaques suggests that LOX-1 has an effect on oxLDL-dependent VSMC proliferation and restenosis [76]. VSMC proliferation is involved in atherogenesis through vascular remodeling and subsequent lesion formation [83–85]. NF-*κ*B- and JNK-signaling pathways are involved in VSMC proliferation, as well [74]. NO is an antioxidant that inhibits VSMC proliferation by reducing the ubiquitin-conjugating enzyme UbcH10 level, which is responsible for the ubiquitination of cell cycle protein. This reduction leads to *G*_0_/*G*_1_ cell cycle arrest, which in turn inhibits VSMC proliferation [86]. As a result of increased ROS production and decreased NO production Ang2 (a LOX-1 and VSMC proliferation inducer) can be upregulated [87, 88].

### 3.4. Influence of LOX-1 on Platelets

LOX-1 is expressed on the surface of human platelets in an activation-dependent manner. The binding of oxLDL to platelets induces thrombus formation by contributing to the ADP-induced activation of fibrinogen receptors such as alpha (IIb) beta (3) and alpha (2) beta (1) integrins [65].

Different pro-inflammatory cytokines like platelet factor 4 (PF4 or CXCL4) and growth factors like platelet-derived growth factor (PDGF) are secreted from the activated platelets [89, 90]. PF4 released from activated platelets facilitates the uptake of oxLDL into the macrophages, which may lead to foam cell formation [91]. PDGF released from activated platelets causes the proliferation and migration of VSMCs [90]. The binding of activated platelets to endothelial surface LOX-1 causes the secretion of endothelin-1, which induces vascular constriction and endothelial dysfunction [92]. Also, the formation of ROS and, subsequently, the inactivation of NO are the next events. As a result, it seems that LOX-1 induces atherosclerosis through binding to oxLDL and activated platelets. It has been demonstrated that aspirin and statins reduce the expression of LOX-1 in platelets [65]. Additionally, LOX-1 and ox-LDL interaction may cause destabilization of plaque through the release of the extracellular MMP inducer CD147 [93].

## 4. Proprotein Convertase Subtilisin/Kexin Type 9 (PCSK9)

### 4.1. Structure, Function, and Signaling

PCSK9, a key protein in lipid metabolism, is the ninth member of the proprotein convertase family and is encoded by the PCSK9 gene in humans on chromosome 1 [94, 95]. PCSK9 is abundantly expressed in the cells of arterial walls, such as endothelium, SMCs, and macrophages, which can regulate vascular homeostasis and atherosclerosis [96–98]. PCSK9 is primarily biosynthesized in the hepatocytes, where it binds LDLR; in addition, it is also expressed in many other tissues, including the kidney, small intestine, lung, pancreas, and brain [99]. In hepatocytes, PCSK9 transports the immature LDLR made in the endoplasmic reticulum to the Golgi membrane, which is glucosidated in the Golgi and then converts to its mature form. Mature LDLR that reaches the cellular level can now be linked to circulating LDL [100–102]. Emma et al. [103] showed that there is a negative correlation between PCSK9 level and liver damage, which means PCSK9 may play a protective role against liver damage. They also showed that PCSK9 levels decreased in hepatic steatosis [103]. In addition, circulating PCSK9 can interfere with the metabolism of triglycerides in heart cells, skeletal muscles, and adipose tissues by degrading very low-density lipoprotein receptors (VLDLR) and apolipoprotein E 2 receptors (ApoER2 or LRP8) [101, 104]. In the atherosclerotic plaque, degradation of LRP-1 (lipoprotein-related protein-1), in which PCSK 9 increases the degradation of this protein, leads to increased expression of tissue factor by ECs and increased pro-inflammatory response by macrophages [105, 106]. The use of NADPH oxidase inhibitors or NF-*κ*B knockout in EC cells has been shown to reduce the production of ROS and LOX-1 and reduce PCSK9 expression [80, 97, 107]. Molecular mechanisms dependent on the regulatory effects of PCSK9 involved in the proliferation and migration of SMCs include the effects of this enzyme on LDLR, LRP-1, VLDLR, and CD36 [108–110]. PCSK9 can proliferate SMCs by mammalian targets of rapamycin [111]. Moreover, PCSK9 is involved in the synthesis of cytokines as well as pro-inflammatory triggers (LPS, ox-LDL, IL-6, IL-1*β*, TNF*α*, and INF-*γ*). In addition, PCSK9 in macrophages can modulate oxLDL uptake and increase foam cells by regulating LOX-1, CD36, and SRA [98, 112–114]. In addition to its role in the degradation of LDL receptors, PCSK9 is associated with an increased risk of coronary artery disease [115]. Based on observational epidemiological studies, plasma levels of CRP are associated with an increased risk of subsequent coronary artery disease [116]. According to a large prospective multicenter study of patients with ACS, those with higher levels of circulating PCSK9 suffer a greater degree of acute-phase inflammation as measured by hs-CRP [117]. Dwivedi et al. [118] observed a relationship between PCSK9 and inflammation. In a mouse sepsis model, PCSK9 overexpression exacerbated lung and liver inflammation, whereas PCSK9 deficiency reduced levels of IL-6 in the blood and reduced organ inflammation. Additionally, some human studies have shown that patients with a PCSK9 LOF allele have significantly lower plasma levels of pro-inflammatory cytokines like TNF-*α*, IL-6, IL-8, and MCP-1 compared with those with a GOF allele [119].

Many polycystic ovary syndrome (PCOS) patients are obese and suffer from atherogenic dyslipidemia, leading to a higher risk of CVD. Recent investigations have elucidated that PCSK9 directly affected ovarian lipid metabolism in PCOS mice. Wang et al. [120] showed that PCSK9 inhibition by alirocumab partly improved lipid profiles and the morphology and function of the ovary in PCOS mice, including dysfunctions associated with endocrine function, follicular growth, and ovulation [112].

### 4.2. PCSK9 and Atherosclerosis

Although the exact role of PCSK9 in the formation of atherosclerotic plaque is unclear, several studies have provided strong evidence for PCSK9 blockade in ischemic heart disease and the development of atherosclerotic plaque through an inflammation-mediated process. The expression of PCSK9 in ECs and SMCs is triggered in pro-inflammatory conditions comprised of ox-LDL, TNF-*α*, IL-1*β*, and LPS [80]. Boyd et al. [121] have found that elevated plasma levels of PCSK9 are associated with systemic inflammatory response syndrome and sepsis. Denis et al. [122] also reported a positive correlation between PCSK9 and atherosclerosis. According to Cheng et al. [123], PCSK9 levels are linearly correlated with the fraction and amount of necrotic core tissue in coronary atherosclerosis, independently of serum LDL cholesterol levels. Notably, PCSK9 inhibitors, either as fully human monoclonal antibodies (evolocumab and alirocumab) or as humanized monoclonal antibodies (bocosizumab), effectively lower LDL-C levels [124]. Treatment with these medicines could significantly reduce major adverse cardiovascular events. In the FOURIER (Further Cardiovascular Outcomes Research with PCSK9 Inhibition in Subjects with Elevated Risk) trial, evolocumab significantly reduced LDL-C levels by 15% for patients with atherosclerotic CVD and LDL-C > 70 mg/dL on statin therapy (HR 0.85; 95% CI, 0.79–0.92; *P* < 0.001) the risk of the primary endpoint (a composite of CV death, MI, stroke, hospitalization for unstable angina, or coronary revascularization) and by 20% (HR 0.80; 95% CI, 0.73–0.88; *P* < 0.001) the risk of the secondary endpoint (a composite of CV death, MI, or stroke) after a median follow-up of 2.2 years [125]. In addition, based on the results of the ODYSSEY OUTCOMES trial, alirocumab therapy reduced the incidence of ACS (the incidence of the primary endpoint (a composite of death from CHD, nonfatal MI, fatal or nonfatal stroke, unstable angina requiring hospitalization)) by 15% in patients on high-intensity statin therapy (HR 0.85; 95% CI, 0.78–0.93; *P* < 0.001). The greatest absolute reduction was observed in the subgroup of patients with the highest baseline LDL-C levels (100 mg/dL) [126]. A study by Bonaca MP indicated that the presence of PCSK9 antibodies was more prevalent in patients with higher CV risk. In fact, patients with peripheral artery disease (PAD) had a greater benefit from evolocumab therapy than patients without PAD [127].

Recent studies have proved that decreasing PCSK9 expression via endogenous RNA interference is a promising therapeutic approach to acutely reducing LDLc and have paved the way for the development of novel PCSK9 lowering agents for the management of severe hypercholesterolemia.

As the first-in-class cholesterol-lowering small interfering RNA (siRNA), Inclisiran (Leqvio®; Novartis) targets triantennary N-acetylgalactosamine carbohydrates (GalNAc). This agent has been approved in the EU in December 2020 as a possible treatment for adults with primary hypercholesterolemia (heterozygous familial and nonfamilial) and mixed dyslipidemia [128].

### 4.3. Possible Cross-Talks between LOX-1, TLR4, and PCSK9

As previously mentioned, NO overproduction can lead to endothelial dysfunction, which is the initial step toward atherosclerosis [129]. OxLDL interferes in modulating the eNOS/inducible nitric oxide synthase (iNOS) machinery. As the oxLDL level rises, so does HMGB1. HMGB1 is a nonhistone DNA-binding protein expressed in most cells, mainly in the nucleus and as a structural component of chromatin. Among the important roles of this protein can be mentioned the participation in the process of DNA replication, recombination, transcription, and repair [130, 131]. HMGB1 can act as a cytokine by being expressed on the plasma membrane or secreted in the extracellular environment and interacts with TLR 2, TLR4, and TLR9 [132]. The interaction of HMGB1 with TLR4 is involved in inducing the release of cytokines such as TNF*α*, IL-1, IL-6, and IL-8 by activated macrophages through the NF-*κ*B pathway [133]. In normal aorta, HMGB1 is expressed in ECs, SMCs, and in CD68 positive macrophages, but in abnormal conditions and in atherosclerotic lesions, HMGB1 expression is increased. Also, strong expression of HMGB1 has been observed in the areas near the necrotic core of atherosclerotic lesions [134]. HMGB1 can activate vascular ECs and thereby lead to the expression and secretion of ICAM-1, VCAM-1, colony-stimulating factor granulocyte, RAGE, and TNF*α* [135, 136]. In response to cellular stress, HMGB1 is released into the extracellular space, studies showed that suppression of HMGB1 leads to reduced LDL transcytosis in human coronary artery EC and vice versa. Studies have shown that this protein plays a role in autophagy and the inhibition of inflammatory nucleosomes, leading to a reduction in inflammation [137, 138].

**Figure 3:**
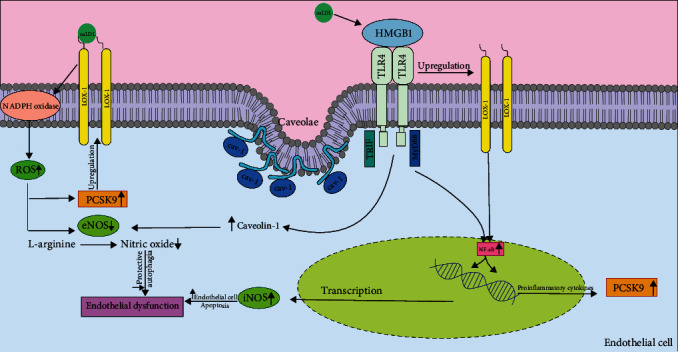
LOX-1, PCSK9, and TLR4 functions in EC. Overall, activated NF-*κ*B upregulates the expression of inflammatory cytokines, such as IL-6, IL-1, and TNF-*α*. As shown, HMGB1 activity causes a decrease in the activity of eNOS. NADPH oxidase is a key mediator of the LOX-1-PCSK9 axis. Upon its activation, there will be an increase in ROS production, which is an inducer for PCSK9 production and further impairment of the function of endothelial cells. ROS, reactive oxygen species; HMGB1, high mobility group box 1 protein; TLR4, Toll-like receptor 4; eNOS, endothelial nitric oxide synthase.

As shown in Figure 3, HMGB1 is involved in TLR4/Caveolin-1 expression pathway in ECs [139]. This pathway downregulates eNOS activity. One important part of TLR4 activation is Caveolin-1 Tyr14 phosphorylation [140]. Furthermore, there is a positive feedback between TLR4 and LOX-1 [141]. Therefore, when TLR4 is activated (by modulating HMGB1), the LOX-1 level also increases. The high level of LOX-1 increases the NF-*κ*B pathway in the nucleus [67]. NF-*κ*B signaling can affect iNOS and causes vascular damage due to an increase in iNOS level (which leads to increased EC apoptosis) and an eNOS level decrease (which leads to reduced protective autophagy); endothelial dysfunction can be initiated [142]. NADPH Oxidase is a key mediator of the LOX-1-PCSK9 axis. In addition, there is a strong correlation between the intracellular ROS concentration and PCSK9 expression [107]. After attaching oxLDL to LOX-1, ROS synthesis is promoted due to the activation of NADPH oxidase. A rise in intracellular ROS leads to upregulation of PCSK9 through a rise in TNF-*α* and reducing eNOS level [143, 144]. Increasing PCSK9 levels cause the degradation of LDLR and consequently leads to LOX-1 upregulation [145]. As mentioned earlier, the reduction in eNOS levels reduces protective autophagy. The combination of increased iNOS and reduced eNOS leads to endothelial dysfunction. More interestingly, RIF and MyD88 are selective adapter molecules involved in TLR4 signaling. According to a study by Liu et al. [146], although the TLR4-MyD88-NF-*κ*B pathway plays an important role in regulating PCSK9, TLR4-TRIF does not. Through the TLR4-MyD88-NF-*κ*B pathway, NF-*κ*B translocation causes the expression of pro-inflammatory genes such as IL-1*β*, IL-18, MCP-1, IL-6, TNF*α*, IL-12, IFN*γ*, and GM-CSF, leading to an upregulation of PCSK9 [146].

## 5. Conclusion

TLR4, LOX-1, and PCSK9 have distinctive roles in atherosclerosis development. Data from clinical and experimental investigations indicate that their inhibitions can be effective to slow the progression of atherosclerosis. Inflammation has a well-distinguished position in atherothrombosis. At present, it is not clearly known how and when to diminish it. Moreover, it is not still conclusive which pharmaceutical interventions are preferred choices. Of note, current findings by important studies like FOURIER, ODYSSEY, and CANTOS are to pave the way for future research and a more robust understanding about lowering lipid levels and also inflammation inhibition.

## Figures and Tables

**Table 1:** Potential ligands for TLR4.

Endogenous ligands	Exogenous ligands
Fibrinogen/fibrin	Fusion protein (RSV)
Heat shock proteins	LPS
Minimally modified LDL	Lipoteichoic acids
OxLDL	Taxol
Heparan sulfate	Mannuronic acid polymers

## Data Availability

Data sharing is not applicable to this article as no datasets were generated or analyzed during the current study.
